# Left behind in the U.S.’ Deep South: Addressing critical gaps in HIV and intimate partner violence prevention efforts targeting Black women

**DOI:** 10.3389/frph.2022.1008788

**Published:** 2022-11-25

**Authors:** Karen A. Johnson, Stefanie Binion, Bernadine Waller, Amber Sutton, Sherron Wilkes, Pamela Payne-Foster, Catherine Carlson

**Affiliations:** ^1^ School of Social Work, The University of Alabama, Tuscaloosa, AL, United States; ^2^ New York State Psychiatric Institute, Columbia University, New York, NY, United States; ^3^ Department of Sociology, Anthropology, and Social Work, Auburn University at Montgomery, Montgomery, AL, United States; ^4^ College of Community Health Sciences, The University of Alabama, Tuscaloosa, AL, United States

**Keywords:** Black women, Deep South, HIV, intimate partner violence, syndemic risk, syndemic protection

## Introduction

Despite repeated calls to action (1), rates of HIV transmission and intimate partner violence (IPV) among low-income, cisgender Black women in the Deep South states of Alabama, Florida, Georgia, Louisiana, Mississippi, South Carolina, and Texas consistently eclipse national averages and disproportionately outpace levels identified among all other women. This high risk group also remains left behind by U.S.’ HIV and IPV research and prevention efforts. They are also projected to be among the most significantly impacted by the recent Supreme Court’s decision to disband Roe v. Wade (2). In view of these risks, and known associations between HIV, IPV, and a lack of access to reproductive services (3), this Opinion serves as an immediate call to action.

In 2019, 8 of 10 U.S. states and 9 out of 10 U.S. metropolitan areas with the highest rates of new HIV diagnoses were in the South, with Deep South states heavily represented among them (4). As many as 9 out of every 10 new HIV cases among women occur in this highly vulnerable group (versus 6 out of 10 cases nationally) (5). In addition, although women are more likely to be tested than men, 90% of new HIV transmissions among women in Deep South states like Alabama and Mississippi (6) are attributed to sexual encounters with male sexual partners, as compared with 77% found elsewhere in the South (4). Disproportionately concentrated among Black women in the Deep South, these higher rates are consistent with earlier findings identifying lower rates of condom negotiation (7) and higher relationship power asymmetries (8). They also consistent with rates of IPV identified in the Deep South that exceed the national average by 12% or higher (9, 10). Yet, there are currently no HIV/IPV prevention interventions that center Deep South specific social and structural factors.

As reflected in Table 1, a narrative literature review of scholarly articles examining “HIV” and “violence” among “Black” or “African American” women in the U.S. reveals that only 12 (or 7%) of a total of 169 articles focused on Black women residing in the Deep South. Of this number, a mere six were written in the past 10 years and none expressly examined the critical intersection of HIV and “intimate partner violence”. In addition, of the 26 of HIV prevention interventions currently in the Centers for Disease Control’s compendium of evidence-based interventions (11), three (or approximately 10%) emanated from the Deep South (12–14). Although one of these emphasized the importance of “sociocultural” and “structural” risks (12), none specifically targeted IPV and/or region-specific differences. While we readily acknowledge these important scholarly contributions, the authors of this Opinion draw attention to these gaps and argue that failing to address them will continue to curtail efforts to end these co-occurring endemics.

**Table 1:** Deep South peer reviewed articles on Black women and the risk for HIV.

Publication date	State (s)	Title	Authors	Population	Methods	Sample size	Risk factors	Protective factors
2004	South Carolina	The changing age of HIV: sexual risk among older African American women living in rural communities	Winningham A, Corwin S, Moore C, Richter D, Sargent R, Gore-Felton C.	African American women aged 50 and older at risk for HIV with at least one sex partner in the past 5 years, and of these, more than half (59.5%) reported at least one sexual risk behavior	Qualitative	(*n* = 181)	• Communication before sex • History of multiple sex partners • History of exchanging sex for money or drugs • Sex with someone who was HIV positive • Sex with someone who was having sex with other women • Sex with someone who was having sex with other men	N/A
2005	Alabama	Domestic violence, sexual ownership, and HIV risk in women in the American Deep South	Lichtenstein B.	HIV-positive African American women who have experienced domestic violence and sexual abuse in the Deep South.	Qualitative	(*n* = 50)	• Women lacked ability to control sexual activities (including condom use) in abusive relationships with HIV-positive men	N/A
2007	Georgia	Factors associated with lack of interest in HIV testing in older at-risk women	Akers A, Bernstein L, Henderson S, Doyle J, Corbie-Smith G	African American women aged ≥50 seeking medical care who were never tested for HIV and women with moderate or high HIV risk factors.	Mixed	(*n* = 514)	• Blood transfusion • Intravenous drug use • Sex with someone who's been in prison • Had sex with someone in Haiti or Africa • Lack of interest in testing • Marital status • Sexual activities • Race	N/A
2008	Georgia	Older women and HIV testing: examining the relationship between HIV testing history, age, and lifetime HIV risk behaviors	Akers AY, Bernstein L, Doyle J, Corbie-Smith G	Older women in a high-prevalence community	Qualitative	(*n* = 514)	• Low HIV knowledge • Low HIV risk perception • Partner with a history of intravenous drug use • Prostitution • Number of sexual partners • Exposure to blood products	N/A
2008	Georgia, Florida	Down low sex, older African American women, and HIV infection	Whyte J 4th, Whyte MD, Cormier E	African American women whose long-term sexual partners had become infected with HIV during extramarital sexual encounters with men.	Qualitative	(*n* = 11)	• Unaware of contracting HIV • Infected by man who had a sexual relationship with another man • Race • Not perceiving risks due to being in long term relationship	N/A
2009	Georgia	Sexual coercion, domestic violence, and negotiating condom use among low-income African American women	Kalichman SC, Williams EA, Cherry C, Belcher L, Nachimson D	Women living in low-income housing developments in Fulton County, Georgia who had engaged in unwanted sex because a male partner threatened to use force or used force to obtain sexual access.	Qualitative	(*n* = 125)	• Sexual coercion • Domestic violence • Substance use • Alcohol abuse	N/A
2016	Georgia	Voices from the unheard: perceptions of HIV among middle class Black women in Atlanta	Heath CD	Middle class Black women with HIV risk perceptions, knowledge, and sexual behaviors.	Qualitative	(*n* = 21)	• Drug users • Women with low educational attainment • Those living in poverty • Women involved in risky heterosexual sex	N/A
2016	Alabama	Structural community factors and sub-optimal engagement in HIV care among low-income women in the Deep South of the USA	Walcott M, Kempf M-C, Merlin JS, Turan JM	Women living with HIV (89% African American) that are affected by structural community factors, such as poverty, poor employment opportunities, limited access to healthcare resources, stigma, transportation challenges and access to illicit substances.	Qualitative	(*n* = 46)	• Economic security • Low educational attainment • Living in poverty • Drug users • Risky heterosexual sex • Adolescents and college students • High incarceration rates of Black men	N/A
2019	Florida	Disparities of HIV risk and PrEP use among transgender women of color in South Florida	Holder CL, Perez-Gilbe HR, Fajardo FJ, Garcia S, Cyrus E	Latina and Black transgender females in South Florida at risk for HIV and living with HIV.	Qualitative	(*n* = 60)	• Unprotected receptive anal sex • Number of sexual partners • Transactional sex	N/A
2020	Florida	Social determinants of depression among older Black women living with HIV	De Oliveira GC, Cianelli R, Villegas N, Solorzano Martinez A, Hires K, Muheriwa SR	Older Black women living with HIV (OBWLH) suffering from depression.	Quantitative	(*n* = 118)	• Lack of condom use • Substance use • Lack of medication adherence	Spirituality Living with a partner
2021	Florida	Predictors end of life discussions among minority older women living with HIV infection	Cianelli R, Villegas N, Lewis-Pierre L, Valdes B, Iriarte E	Black and 19 Hispanic women aged 50 + living with HIV	Qualitative	(*n* = 138)	N/A	Religion Living with partner or caregiver
2022	Florida	Exploring the psychosocial impact of living with HIV on minority older women	Cianelli R, Villegas N, Oliveira GD, Sailsman S, Montano NP, Martinez AS, et al.	Older minority women living with HIV.	Qualitative	(*n* = 28)	• Unprotected sexual intercourse • Natural weakening of the immune system • Having casual sex partners	Social support Religion/spirituality

### The high cost of inaction

The costs associated with this continued neglect are sobering and anticipated to see sharp inclines. Although individuals in the Deep South make up only 29% of the total U.S. population, in 2019 alone, 47% of deaths in the U.S. attributed to HIV were concentrated in this region of the country. In addition, despite recent encouraging declines in the rate of new HIV diagnoses across the U.S. due to treatment advances such as PrEP and PEP, this decrease is markedly slower in the Deep South due in part to concentrated social and structural barriers endemic to this region of the U.S. (15).

The projected increase in HIV and IPV attributed directly to the recent retrenchment in sexual and reproductive services is of particular concern in the Deep South states given what scholars have also characterized as HIV and IPV risks specific to and/or exacerbated by residing in this region of the country. Although significantly understudied, these “Deep South-specific” or “Deep South-exacerbated” risks are noted to include (1) among the highest levels of (16) conservatism (e.g., religious, political, patriarchal gender-role) in the U.S. (17); (2) heavily concentrated poverty that eclipses rates found in developing nations and elsewhere in the U.S. (18); (3) health, behavioral health, Wi-Fi, and transportation deserts (19); and (4) disabling self, faith-based, interpersonal, systemic, and community stigma (20). Not enough is known however regarding how these region-specific and/or region-exacerbated factors may combine in a syndemic-like manner and/or may mediate individual and/or interpersonal risks. Syndemics is defined as two or more inextricable epidemics that work synergistically to significantly impact the overall health status of a population (21). In addition to increasing the risk for adverse outcomes, for a true syndemic to exist, these linked epidemics must also amplify each other, leading to worsening outcomes. Perhaps the most well documented syndemic is the SAVA syndemic which posits that poor HIV/AIDS outcomes are highly correlated with substance misuse and IPV among impoverished populations (22). A handful of HIV and IPV studies have also drawn attention to the importance of examining social, structural and cultural risks (alongside extreme poverty) (23, 24). None to date however, have examined Deep South-specific drivers of syndemic outcomes. This gap may be attributed to the lack of large-scale quantitative studies that examine HIV/IPV risk co-occurrence among Black women in this region of the U.S. These gaps notwithstanding, a direct causal link (25) and bidirectional associations (26) has been identified between HIV and IPV. Scholars have also identified salient differences in forms of HIV risks (e.g., engaging in casual and survival sex versus having concurrent sex partners) and IPV Black women experience based on the geographical location, culture, and norms (27). However, how factors may separately or together mediate HIV/IPV remains unclear.

Significant gaps also remain regarding possible syndemic-like protections unique to and/or amplified by living in the Deep South, such as faith and southern culture, despite literature pointing to the incredible saliency of both in the lives of Black women in the South (28, 29). Instead, HIV and IPV prevention literature has been disproportionately deficit in focus and region-specific protective factors remain vastly under-explored. As also denoted in Table 1, of the 12 articles identified in the narrative review, three examined population-specific protective factors (e.g., “living with partner or caregiver, social support, religion”). None however examined how factors heavily concentrated in the region may operate structurally to provide protection from transmission. This, despite the fact that the church remains one of the strongest and widely utilized sources of support for individuals, Black or otherwise, residing throughout the Deep South and faith-based organizations have served as critical partners in delivering social services, health-related, and prevention interventions (28). Scholars have also pointed to a “culture of honor” in the Deep South characterized by strong levels of gender roles and family cohesion (30). These highly patriarchal roles that characterize systemic approaches in this area of the country may further dissuade women’s help-seeking efforts (31). Little to no research exists however regarding if and how this concept impacts the lives of low-income cisgender Black women in the Deep South through social and structural mechanisms.

Fundamental to improving this research is including Black women in the Deep South in the research process. Community-partnered approaches employed throughout the continuum of the research process is a proven mechanism for increasing participation among traditionally excluded, understudied and underrepresented populations (32). Unlike community-based research where power differentials between the academic research community may erode trust, community partnered approaches rely upon shared power which often translates into higher rates of participation among underserved communities (32). Even so, opportunities to engage low-income Black women in the Deep South as full health equity partners in mapping Deep South specific HIV/IPV risks and protections remain woefully underleveraged.

## Discussion

To close these critical gaps in HIV and IPV prevention research and interventions, and counter anticipated increases stemming from Roe vs. Wade, we recommend that urgent consideration be given to the following:

### Health equity partnerships with Black women

Black women residing in the Deep South should be involved in deciding what research and praxis is needed, conceptualizing and framing research questions, determining the theoretical lens and potential interventions. Instead, they have at times been categorized as especially difficult to reach and engage (33) and are distrustful of the medical system and/or evidence-based interventions (34). Without equitable sharing of power and voice in all phases of research and intervention design, it is likely that these efforts, should they be prioritized, will replicate existing deficit-based scholarship and interventions. This gap in engaging Black women in the Deep South as health equity partners stands in contrast to the U.S.’ Ending the HIV Epidemic (EHE) strategic priorities. Among other key strategies set forth by the U.S.’ Health and Human Services’ (HHS) Office of Infectious Disease and HIV/AIDS Policy is a commitment to “plan(ning), design(ing), and deliver(ing) HIV prevention and care services” that are fully reflective of the needs of localities (35), the absence of which may result in a continued “stall(ing) of progress” in eliminating new HIV diagnoses.

### Syndemic explorations: Structural risks and protections

Additional quantitative and multi-methods studies are also needed to advance the understanding of how risks and/or protections may combine “syndemically” to drive or mitigate risks. We recommend that every consideration be given to how region-specific social and structural differences may combine with individual and interpersonal factors to increase or decrease adverse outcomes. Deep South faith and culture have yet to be fully understood or leveraged. It is essential that we understand how they may uniquely impact risks (both positively and negatively) from the vantage point of low-income Black women in the Deep South, and how they should and should not be leveraged. Advancing research in this regard is consistent with models and frameworks such as the social ecological model of prevention (36) and social determinants of health (37), that acknowledges the critical role social and structural dimensions of risk (and resilience) may have on health outcomes. Despite literature that points to the determinative role that “place” plays in health and behavioral health outcomes (38), and overall well-being, to date none currently model region-specific dimensions of risks and resilience.

### Exploration of implementation science implications

Lastly, alongside efforts to better understand the impact of Deep South-specific social and structural risks on HIV and IPV outcomes, we recommend that research be conducted regarding what impact these region-specific risks may have on efforts to implement and scale prevention interventions. A growing number of implementation science frameworks such as the Health Equity Implementation Framework (HEIF) (39) suggest that “societal” and “sociopolitical” domains are determinative of implementation science outcomes. Through the lens of HEIF, a critical next step is to examine if and how Deep South specific risks and protections such as “stigma”, “conservatism”, “faith” and “culture” may permeate organizations, faith-based or otherwise. By extension, it will also be important to examine if and how the organization itself (e.g., formal and informal policies, procedures, and culture) and/or organizational personnel in highly conservative and stigmatized social, political, and cultural environments may intentionally or unintentionally work to undermine HIV and IPV implementation efforts.
